# Distributed Kalman Filter with Maximum Correlation Entropy Criterion and Consensus Weighted Term Fusion

**DOI:** 10.3390/e28080941

**Published:** 2026-08-21

**Authors:** Xiaoliang Feng, Zhouliner Gao, Teng Liu

**Affiliations:** School of Electrical and Energy Engineering, Shanghai Dianji University, Shanghai 201306, China; fengxl2002@163.com (X.F.); liuteng2313@163.com (T.L.)

**Keywords:** distributed filtering, consensus error, maximum correntropy criterion, consensus weighting, state estimation

## Abstract

Distributed maximum correntropy Kalman filters improve robustness to non-Gaussian noise, but existing variants generally introduce consensus through average or weighted fusion without explicitly separating the innovation residual from the state-disagreement residual in a dimensionally consistent objective. This paper proposes a Distributed Maximum Correntropy Kalman Filter with Innovation and Consensus Weighting Terms (DMCKF-IW-CWT). The innovation and consensus residuals are normalized separately and mapped by Gaussian kernels, after which the resulting information matrices are incorporated into a fixed-point local update. Posterior covariance intersection (CI) is then used to fuse neighboring estimates without requiring the unavailable cross-covariances. A sufficient contraction condition is given for the fixed-point iteration. In a five-node benchmark with 500 independent Monte Carlo runs and 1000 sampling steps, the proposed method obtains overall, transient, and steady-state MAEs of 0.172210, 0.188271, and 0.168195, respectively, corresponding to reductions of 0.254%, 0.526%, and 0.178% relative to DMCKF-W; the paired 95% confidence intervals of all three differences remain below zero. The consensus RMS is further reduced by 5.371%. Additional tests involving five noise families, packet loss and communication noise, a four-state nonlinear model, and systems with up to eight states and twenty nodes confirm the numerical convergence and extensibility of the framework.

## 1. Introduction

Distributed fusion estimation does not rely on a central fusion node and is therefore widely applicable to target tracking, communication networks, and power systems [1,2,3,4,5,6]. In sensor networks, consensus fusion represents one of the most commonly utilized distributed fusion strategies, which aims to drive the estimates of each node toward consistency and achieve the theoretically optimal estimate. Generally, the primary consensus strategies adopted in distributed estimation include average consensus, weighted consensus based on covariance intersection fusion, and weighted consensus based on correntropy, among others [7,8,9,10].

For linear Gaussian systems, distributed Kalman filters based on average consensus [7] and distributed Kalman filters based on covariance intersection fusion [11] have been developed. For nonlinear Gaussian systems, Battistelli and Chisci [12] proposed a Distributed Extended Kalman Filter (DEKF) based on a weighted consensus strategy. However, due to its inherent disadvantages such as instability and low-order accuracy, Li and Jia [13] developed the statistical linear error propagation method to derive a pseudo-measurement matrix, proposing a Distributed Unscented Kalman Filter (DUKF). Based on the DUKF framework, Li et al. [14] designed a DUKF algorithm based on weighted average consensus. Nonetheless, when the system state dimension is large, the DUKF algorithm may suffer from filter divergence. To address high-dimensional nonlinear state estimation problems, Chen et al. [15] proposed a cubature Kalman filter based on weighted average consensus. This filter retains the advantages of distributed filtering, such as scalability and robustness to single-node failures, while also offering the high accuracy and strong stability of the cubature Kalman filter. All the aforementioned algorithms are designed based on the minimum mean square error criterion. Since the performance metrics of these algorithms are functions of the second-order moments of the estimation error, these distributed filtering methods perform well when dealing with state estimation problems corrupted by Gaussian noise but exhibit poor performance when confronting distributed filtering problems under non-Gaussian noise interference.

Robust distributed estimation under nonlinear and non-Gaussian conditions has also received considerable attention. Samadi et al. [16] introduced a distributed particle filter based on average consensus. Since particle filters require a large number of particles for local filtering at each sensor node, leading to high computational costs, Wang et al. [17] incorporated information learning theory to propose a Distributed Maximum Correntropy Kalman Filter (DMCKF). Its core contribution is the extension of the Maximum Correntropy Kalman Filter (MCKF) to a distributed framework by introducing a new gain matrix. Wang et al. [18] proposed a distributed maximum correntropy linear and nonlinear information filter, whose primary concept is to design a distributed maximum correntropy information filter using an average consensus strategy based on the information form of centralized MCKF to approximate the centralized counterpart, which was then extended to nonlinear systems. Hu and Chen [19] developed a DMCKF algorithm based on covariance intersection fusion, which primarily improves estimation performance by utilizing matrix weights derived from the maximum correntropy criterion. This algorithm requires communicating with neighbors only once per sampling period, making it more efficient in low-bandwidth communication scenarios compared to standard DMCKF algorithms.

Based on the DMCKF framework, Wang et al. [10] proposed a weighted consensus strategy based on a correntropy loss function (DMCKF-W-MCC). The loss function of this method considers not only the estimated states of the nodes but also the observations of neighboring nodes, thereby mitigating state discrepancies among nodes. Furthermore, the distributed minimum error entropy Kalman filter proposed in Feng et al. [20] incorporates the minimum error entropy criterion into the Distributed Kalman Filter (DKF), resolving the distributed information fusion problem in wireless sensor networks under non-Gaussian noise. The aforementioned distributed algorithms typically utilize second-order statistical characteristics to measure the consensus problem, whereas research on measuring consensus issues via high-order statistics remains scarce. Multi-source information fusion provides a general framework for combining heterogeneous sensor information [21]. From an information-theoretic perspective, correntropy provides a robust similarity measure for non-Gaussian data [22] and has been applied to robust estimation problems, including UWB positioning [23] and fixed-point smoothing under non-Gaussian environments [24].

Despite these developments, the roles of innovation robustness and network consensus are not fully separated in existing DMCKF variants. DMCKF extends MCKF to distributed estimation, DMCKF-A applies average consensus, and DMCKF-W uses covariance-based weights, but these mechanisms mainly rely on averaging or second-order uncertainty information. DMCKF-W-MCC introduces correntropy-based weighting of neighboring information; however, its local criterion does not explicitly isolate a normalized consensus-disagreement residual from the innovation residual, and covariance fusion remains difficult when neighboring posterior estimates are correlated. Consequently, a dimensionally consistent high-order consensus metric and a fusion rule that remains valid under unknown cross-covariances are still required.

This paper addresses the above gap through the following contributions. First, we construct a dual-residual maximum correntropy criterion by separating the whitened innovation residual and the consensus-disagreement residual, with their dimensions and normalization factors stated explicitly. Second, a fixed-point local estimator is derived and combined with posterior covariance intersection, so that neighboring posterior estimates can be fused without assuming that their estimation errors are independent. A sufficient contraction-based convergence condition is provided for the fixed-point recursion, and a conditional mean-square boundedness result is established under explicit uniform boundedness and covariance conditions. Third, we conduct a paired Monte Carlo evaluation using common random realizations, confidence intervals, transient and steady-state metrics, covariance-consistency indicators, communication impairments, multiple noise families, nonlinear measurements, and higher-dimensional networks. Related studies have established Kalman filtering for descriptor systems and for fractional-order singular systems [25,26,27], providing a relevant basis for considering such model classes. Accordingly, the modular separation between the model-specific local prediction stage and the subsequent consensus-weighted update/CI fusion suggests a possible future extension of the present framework to descriptor or fractional-order singular models. This is a prospective extension rather than an established property of the proposed method, and model-specific derivation together with verification of the convergence and boundedness conditions would be required. The present study only verifies extensibility through a nonlinear range-only model and linear systems with up to eight states and twenty nodes.

## 2. Problem Formulation

Consider a sensor network consisting of *N* nodes. The distributed dynamic system can be described as:

(1) $$x_{k}=F_{k}x_{k-1}+w_{k,k-1}$$

(2) $$y_{k}^{i}=H_{k}^{i}x_{k}+v_{k}^{i}$$

where *i* = 1,2,…, *N* denotes the sensor index. The state is *p*-dimensional, and each node supplies a *q*-dimensional measurement. The corresponding state-transition and node measurement matrices appear in (1) and (2). The process and measurement noises are zero mean, with covariance matrices specified in (3). They are mutually uncorrelated, and measurement noises from distinct nodes are also uncorrelated:

(3) $$\{\begin{array}{c} E\left[w_{k,k-1}w_{l,l-1}^{T}\right]=Q_{k,k-1}\delta_{kl},\\ E\left[v_{k}^{i}{\left(v_{l}^{j}\right)}^{T}\right]=R_{k}^{i}\delta_{ij}\delta_{kl},\\ E\left[w_{k,k-1}{\left(v_{l}^{j}\right)}^{T}\right]=0 \end{array}$$

where E[·] denotes mathematical expectation, and $\delta_{ab}$ denotes the Kronecker delta, defined as:

(4) $$\delta_{ab}=\{\begin{array}{c} 1,a=b\\ 0,a\neq b \end{array}$$

When the process and measurement noises are non-Gaussian, DMCKF-W-MCC [10] improves robustness by applying correntropy-based weights to neighboring information. Nevertheless, the innovation residual and the consensus discrepancy are not represented as two explicitly separated, dimensionally normalized components of the local objective. Moreover, posterior estimates exchanged in a distributed network are generally correlated, so a covariance update based on zero cross-covariance assumptions may become inconsistent. The next section therefore introduces a dual-residual correntropy criterion and combines the resulting local update with covariance intersection, which does not require the cross-covariances among neighboring posterior estimates.

## 3. Distributed Kalman Filter Based on Maximum Correntropy Criterion with Consensus Weighting Terms

For the distributed linear system described by (1) and (2), assume that at time *k* − 1, the state estimate of node *i* is known as ${\hat{x}}_{k-1|k-1}^{i}$, and its corresponding estimation error covariance matrix is $P_{k-1|k-1}^{i}$. Furthermore, assume that the state estimate of the neighboring node *j* at time *k*−1 is known as ${\hat{x}}_{k-1|k-1}^{j}$, and its estimation error covariance matrix is $P_{k-1|k-1}^{j}$.

The state prediction and the state prediction error covariance matrix of node *i* are given by:

(5) $${\hat{x}}_{k|k-1}^{i}=F_{k}{\hat{x}}_{k-1|k-1}^{i}$$

(6) $$P_{k|k-1}^{i}=F_{k}P_{k-1|k-1}^{i}F_{k}^{T}+Q_{k,k-1}$$

Similarly, the state prediction and the corresponding state prediction error covariance matrix of the neighboring node *j* are given by:

(7) $${\hat{x}}_{k|k-1}^{j}=F_{k}{\hat{x}}_{k-1|k-1}^{j}$$

(8) $$P_{k|k-1}^{j}=F_{k}P_{k-1|k-1}^{j}F_{k}^{T}+Q_{k,k-1}$$

For node *i*, stack the local prior relation with the measurements received from its inclusive measurement neighborhood. Under ideal communication, this set contains node *i* and all of its neighbors; under packet loss, it contains node *i* and the successfully received neighboring measurements. The augmented model is:

(9) $$\left[\begin{array}{c} {\hat{x}}_{k|k-1}^{i}\\ y_{k}^{J_{i}} \end{array}\right]=\left[\begin{array}{c} I_{P}\\ H_{k}^{J_{i}} \end{array}\right]x_{k}+\tau_{k}^{i}$$

where the stacked measurement, measurement matrix, noise vector, and block-diagonal covariance are:

(10) $$\begin{array}{c} \tau_{k}^{i}=col\;\left({\hat{x}}_{k|k-1}^{i}-x_{k},\;v_{k}^{j}:j\in J_{i}\right),\\ y_{k}^{J_{i}}=col\;\left(y_{k}^{j}:j\in J_{i}\right),\;H_{k}^{J_{i}}=col\;\left(H_{k}^{j}:j\in J_{i}\right),\\ R_{k}^{J_{i}}=blkdiag\;\left(R_{k}^{j}:j\in J_{i}\right) \end{array}$$

Lower-triangular Cholesky factors are used throughout, so that each covariance equals its factor multiplied by its transpose:

(11) $$P_{k|k-1}^{i}=B_{P,k}^{i}{\left(B_{P,k}^{i}\right)}^{T},\;\;R_{k}^{J_{i}}=B_{R,k}^{J_{i}}{\left(B_{R,k}^{J_{i}}\right)}^{T}$$

(12) $$B_{k}^{i}=blkdiag\;\left(B_{P,k}^{i},B_{R,k}^{J_{i}}\right)$$

Premultiplying (9) by the inverse of the block factor in (12) gives the whitened augmented quantities:

(13) $$D_{k}^{i}={\left(B_{k}^{i}\right)}^{-1}\left[\begin{array}{c} {\hat{x}}_{k|k-1}^{i}\\ y_{k}^{J_{i}} \end{array}\right]$$

(14) $$W_{k}^{i}={\left(B_{k}^{i}\right)}^{-1}\left[\begin{array}{c} I\\ H_{k}^{J_{i}} \end{array}\right]$$

(15) $$e_{I,k}^{i}=D_{k}^{i}-W_{k}^{i}x_{k}={\left(B_{k}^{i}\right)}^{-1}\tau_{k}^{i}$$

Let *J_i_* denote the inclusive measurement neighborhood, let *n*_i_ be its cardinality, and let *L_i_ = p + n_i_q*. Hence the augmented data and innovation vectors contain *p* prior components and *n_i_q* measurement components. The set *N_i_* contains the neighboring predicted states successfully received by node *i*. The two sets are distinct because the measurement stack includes the local sensor, whereas the consensus target uses neighboring predictions.

The consensus residual is the difference between the current fixed-point state and the covariance-weighted prediction target. The neighboring predictions are received once per sampling instant and remain fixed during the local iteration:

(16) $${\overset{‾}{x}}_{c,k}^{i}=\sum_{j\in N_{i}}C_{k}^{ij}{\hat{x}}_{k|k-1}^{j}$$

where:

(17) $$C_{k}^{ij}=\Lambda_{k}^{i}\beta_{k}^{ij}{\left(P_{k|k-1}^{j}\right)}^{-1}$$

(18) $$\Lambda_{k}^{i}={\left[{\sum_{r\in N_{i}}\beta_{k}^{ir}\left(P_{k|k-1}^{r}\right)}^{-1}\right]}^{-1}$$

(19) $$\beta_{k}^{ij}=\frac{1/tr\left(P_{k|k-1}^{j}\right)}{\sum_{r\in N_{i}}\left(1/tr\left(P_{k|k-1}^{r}\right)\right)}$$

Two Gaussian kernels distinguish measurement robustness from inter-node agreement. The innovation bandwidth controls the attenuation of augmented innovation outliers, whereas the consensus bandwidth controls sensitivity to the whitened state disagreement. The squared bandwidth factors in (20) cancel the corresponding Gaussian-kernel derivative factors, and the consensus-strength parameter controls the relative contribution. The dimension-normalized objective is:

(20) $$J_{k}^{i}\left(x\right)=\frac{1}{p+L_{i}}\left[\lambda_{c}\sigma_{c}^{2}\sum_{l=1}^{p}G_{\sigma_{c}}\left(e_{c,k}^{i}\left(l\right)\right)+\sigma_{I}^{2}\sum_{l=1}^{L_{i}}G_{\sigma_{I}}\left(e_{I,k}^{i}\left(l\right)\right)\right],\;L_{i}=p+n_{i}q$$

Because the consensus residual contributes *p* scalar components and the innovation residual contributes *L_i_* components, the common normalization factor is *p + L_i_*. The two unnormalized components are:

(21) $$\{\begin{array}{c} J_{c,k}^{i}=\sum_{l=1}^{p}G_{\sigma_{c}}\left(e_{c,k}^{i}\left(l\right)\right),\\ J_{I,k}^{i}=\sum_{l=1}^{L_{i}}G_{\sigma_{I}}\left(e_{I,k}^{i}\left(l\right)\right) \end{array}$$

The local estimate maximizes (20):

(22) $${\hat{x}}_{k}^{i}=\underset{x\in R^{p}}{arg\;max}\;J_{k}^{i}\left(x\right)$$

At each fixed-point iteration, the Gaussian weights and the dimension-normalization scale are evaluated at the previous iterate and then held fixed while solving the weighted surrogate. Differentiating that surrogate gives (23). The effective consensus matrix includes the consensus-strength and covariance-shape normalization in (45), while the innovation matrix uses (28)–(30). The common positive normalization factor cancels from the stationarity equation:

(23) $$\frac{\partial J_{k}^{i}}{\partial x}=-\frac{1}{p+L_{i}}\left[{\tilde{\Omega }}_{c,k}^{i}\left(x-{\overset{‾}{x}}_{c,k}^{i}\right)+{\left(W_{k}^{i}\right)}^{T}\Omega_{I,k}^{i}\left(W_{k}^{i}x-D_{k}^{i}\right)\right]=0$$

Rearranging the stationarity equation yields the following dimensionally compatible normal equation:

(24) $$\left[{\tilde{\Omega }}_{c,k}^{i}+{\left(W_{k}^{i}\right)}^{T}\Omega_{I,k}^{i}W_{k}^{i}\right]x={\left(W_{k}^{i}\right)}^{T}\Omega_{I,k}^{i}D_{k}^{i}+{\tilde{\Omega }}_{c,k}^{i}{\overset{‾}{x}}_{c,k}^{i}$$

Because the matrix on the left-hand side is positive definite under the assumptions stated in Section 4.3, the optimal local solution is:

(25) $$x={\left[{\tilde{\Omega }}_{c,k}^{i}+{\left(W_{k}^{i}\right)}^{T}\Omega_{I,k}^{i}W_{k}^{i}\right]}^{-1}\left[{\left(W_{k}^{i}\right)}^{T}\Omega_{I,k}^{i}D_{k}^{i}+{\tilde{\Omega }}_{c,k}^{i}{\overset{‾}{x}}_{c,k}^{i}\right]$$

Both information matrices depend on the innovation and consensus residuals and therefore on the current state. Consequently, (25) defines a nonlinear fixed-point equation. With the weights evaluated at the previous iterate, the implemented recursion is:

(26) $$x_{k,t}^{i}=F_{i,k}\left(x_{k,t-1}^{i}\right)=A_{i,k}^{-1}\left(x_{k,t-1}^{i}\right)b_{i,k}\left(x_{k,t-1}^{i}\right)$$

In (23) and (26), the untilded consensus matrix denotes the raw diagonal Gaussian-kernel weights, whereas the tilded matrix denotes the dimensionally scaled effective consensus information used in the fixed-point update. This distinction is maintained throughout the remainder of the paper.

To avoid numerical singularity when a residual is very large, a small positive lower bound is imposed on the Gaussian-kernel weights during the fixed-point implementation. Specifically, the stabilized kernel weight is defined as:

(27a) $${\overset{‾}{G}}_{\sigma }\left(e\right)=max\left\{G_{\sigma }\left(e\right),\varepsilon_{g}\right\},\varepsilon_{g}>0$$

The original Gaussian kernel is retained in the maximum correntropy objective in (20). The lower-bounded kernel is used only in the numerical weight matrices of the fixed-point recursion. Since is chosen to be very small, this treatment acts as a numerical regularization while preventing the information matrix from becoming singular.

Where the raw Gaussian kernel matrices are:

(27b) $$\Omega_{c,k}^{i}=diag\left({\overset{‾}{G}}_{\sigma_{c}}\left(e_{c,k}^{i}\left(1\right)\right),\ldots ,{\overset{‾}{G}}_{\sigma_{c}}\left(e_{c,k}^{i}\left(p\right)\right)\right)$$

(28) $$\Omega_{I,k}^{i}=blkdiag\;\left(\Omega_{x,I,k}^{i},\Omega_{y,I,k}^{i}\right)$$

(29) $$\Omega_{x,I,k}^{i}=diag\left({\overset{‾}{G}}_{\sigma_{I}}\left(e_{I,k}^{i}\left(1\right)\right),\ldots ,{\overset{‾}{G}}_{\sigma_{I}}\left(e_{I,k}^{i}\left(p\right)\right)\right)$$

(30) $$\Omega_{y,I,k}^{i}=diag\;\left({\overset{‾}{G}}_{\sigma_{I}}\left(e_{I,k}^{i}\left(p+1\right)\right),\ldots ,{\overset{‾}{G}}_{\sigma_{I}}\left(e_{I,k}^{i}\left(L_{i}\right)\right)\right)$$

(31) $${\hat{x}}_{k|k}^{i,loc}={\left[{\left({\overset{‾}{P}}_{k}^{i}\right)}^{-1}+S_{k}^{i}+{\tilde{\Omega }}_{c,k}^{i}\right]}^{-1}\left[{\left({\overset{‾}{P}}_{k}^{i}\right)}^{-1}{\hat{x}}_{k|k-1}^{i}+Z_{k}^{i}+{\tilde{\Omega }}_{c,k}^{i}{\overset{‾}{x}}_{c,k}^{i}\right]$$

The augmented data and innovation vectors have *L_i_* components; the augmented model matrix has *L_i_* rows and *p* columns; the innovation-weight matrix is *L_i_*-by-*L_i_*; and the effective consensus, prior-information, measurement-information, covariance, and consensus-weight matrices are *p*-by-*p*. The information vector has *p* components. Thus, all products and sums in (23)–(37) are dimensionally compatible.

(32) $$M_{k}^{i}={\left({\overset{‾}{P}}_{k}^{i}\right)}^{-1}+{\overset{‾}{S}}_{k}^{i}$$

(33) $${\overset{‾}{P}}_{k}^{i}=B_{P,k}^{i}{\left(\Omega_{x,I,k}^{i}\right)}^{-1}{\left(B_{P,k}^{i}\right)}^{T}$$

(34) $$S_{k}^{i}=\sum_{j\in J_{i}}{\left(H_{k}^{j}\right)}^{T}{\left({\overset{‾}{R}}_{k}^{j}\right)}^{-1}H_{k}^{j}$$

(35) $$Z_{k}^{i}=\sum_{j\in J_{i}}{\left(H_{k}^{j}\right)}^{T}{\left({\overset{‾}{R}}_{k}^{j}\right)}^{-1}y_{k}^{j}$$

(36) $${\overset{‾}{R}}_{k}^{j}=B_{R,k}^{j}{\left(\Omega_{y,I,k}^{j}\right)}^{-1}{\left(B_{R,k}^{j}\right)}^{T}$$

(37) $${\hat{x}}_{k|k}^{i,loc}={\hat{x}}_{k|k-1}^{i}+K_{k}^{i}\left[Z_{k}^{i}-S_{k}^{i}{\hat{x}}_{k|k-1}^{i}+{\tilde{\Omega }}_{c,k}^{i}\left({\overset{‾}{x}}_{c,k}^{i}-{\hat{x}}_{k|k-1}^{i}\right)\right]$$

Equation (37) is the local IW-CWT posterior update. Because local posterior errors can be correlated through common process information and prior exchanges, node *i* subsequently fuses the local posterior information pairs by covariance intersection:

(38a) $$P_{k|k}^{i}={\left[\sum_{j\in J_{i}}\omega_{ij}{\left(P_{k|k}^{j,loc}\right)}^{-1}\right]}^{-1}$$

(38b) $${\hat{x}}_{k|k}^{i}=P_{k|k}^{i}\sum_{j\in J_{i}}\omega_{ij}{\left(P_{k|k}^{j,loc}\right)}^{-1}{\hat{x}}_{k|k}^{j,loc}$$

The CI weights are nonnegative and sum to one over the available local and neighboring posteriors. The implementation normalizes covariance-trace weights. Equations (38a,b) do not require posterior cross-covariances and replace the direct additive prediction-state correction used in the earlier draft.

The proposed DMCKF-IW-CWT algorithm can be summarized as follows:

Step 1: Select the innovation bandwidth, consensus bandwidth, consensus-strength coefficient, and a positive stopping threshold. Initialize the state estimate and covariance of each node.

Step 2: Compute the predicted states and prediction error covariance matrices for node *i* and its neighboring nodes *j* by utilizing (5)–(8), respectively.

Step 3: Set the initial iteration step to *t* = 1, and let ${\hat{x}}_{k|k,0}^{i}={\hat{x}}_{k|k-1}^{i}$, where ${\hat{x}}_{k|k,t}^{i}$ denotes the filtered estimate of the *i*-th node at the *t*-th fixed-point iteration.

Step 4: Compute the lower-triangular Cholesky factors of the predicted covariance and the stacked measurement covariance according to (11) and (12).

Step 5: Compute the consensus weights, the CI covariance bound, and the covariance-weighted prediction target. Form the consensus whitening covariance according to (48), evaluate the stabilized Gaussian kernel weights defined in (27a), and construct the effective consensus information matrix according to (45). Starting from the predicted state, apply the fixed-point recursion in (26), with the implementation quantities defined in (39)–(49). Neighboring predictions are communicated once per sampling instant and reused during the iteration.

(39) $${\hat{x}}_{k|k,t}^{i}={\hat{x}}_{k|k-1}^{i}+K_{k,t-1}^{i}\left[Z_{k,t-1}^{i}-S_{k,t-1}^{i}{\hat{x}}_{k|k-1}^{i}+{\tilde{\Omega }}_{c,k,t-1}^{i}\left({\overset{‾}{x}}_{c,k}^{i}-{\hat{x}}_{k|k-1}^{i}\right)\right]$$

Where:

(40) $$K_{k,t-1}^{i}={\left[{\left({\overset{‾}{P}}_{k,t-1}^{i}\right)}^{-1}+S_{k,t-1}^{i}+{\tilde{\Omega }}_{c,k,t-1}^{i}\right]}^{-1}$$

(41) $${\overset{‾}{P}}_{k,t-1}^{i}=B_{P,k}^{i}{\left(\Omega_{x,I,k,t-1}^{i}\right)}^{-1}{\left(B_{P,k}^{i}\right)}^{T}$$

(42) $$S_{k,t-1}^{i}=\sum_{j\in J_{i}}{\left(H_{k}^{j}\right)}^{T}{\left({\overset{‾}{R}}_{k,t-1}^{j}\right)}^{-1}H_{k}^{j}$$

(43) $$\Omega_{x,k,t-1}^{i}=diag\left({\overset{‾}{G}}_{\sigma_{I}}\left(e_{I,k,t-1}^{i}\left(1\right)\right),\ldots ,{\overset{‾}{G}}_{\sigma_{I}}\left(e_{I,k,t-1}^{i}\left(p\right)\right)\right)$$

(44) $$Z_{k,t-1}^{i}=\sum_{j\in J_{i}}{\left(H_{k}^{j}\right)}^{T}{\left({\overset{‾}{R}}_{k,t-1}^{j}\right)}^{-1}y_{k}^{j}$$

(45) $${\overset{‾}{R}}_{k,t-1}^{j}=B_{R,k}^{j}{\left(\Omega_{y,I,k,t-1}^{j}\right)}^{-1}{\left(B_{R,k}^{j}\right)}^{T}$$

(46) $${\tilde{\Omega }}_{c,k,t-1}^{i}=\lambda_{c}\frac{tr\left[{\left({\overset{‾}{P}}_{k,t-1}^{i}\right)}^{-1}+S_{k,t-1}^{i}\right]}{tr\left[{\left(R_{c,k}^{i}\right)}^{-1}\right]}{\left(B_{c,k}^{i}\right)}^{-T}\Omega_{c,k,t-1}^{i}{\left(B_{c,k}^{i}\right)}^{-1}$$

(47) $$\Omega_{c,k,t-1}^{i}=diag\left({\overset{‾}{G}}_{\sigma_{c}}\left(e_{c,k,t-1}^{i}\left(1\right)\right),\ldots ,{\overset{‾}{G}}_{\sigma_{c}}\left(e_{c,k,t-1}^{i}\left(p\right)\right)\right)$$

(48) $$\Omega_{y,I,k,t-1}^{i}=diag\left({\overset{‾}{G}}_{\sigma_{I}}\left(e_{I,k,t-1}^{i}\left(p+1\right)\right),\ldots ,{\overset{‾}{G}}_{\sigma_{I}}\left(e_{I,k,t-1}^{i}\left(L_{i}\right)\right)\right)$$

(49) $$e_{c,k,t-1}^{i}={\left(B_{c,k}^{i}\right)}^{-1}\left({\hat{x}}_{k|k,t-1}^{i}-{\overset{‾}{x}}_{c,k}^{i}\right),\;R_{c,k}^{i}=\kappa_{c}\left(P_{k|k-1}^{i}+\Lambda_{k}^{i}\right)$$

Step 6: Stop the network fixed-point iteration when the largest node-wise relative change satisfies:

(50) $${max}_{i=1,\ldots ,N}\frac{∥{\hat{x}}_{k|k,t}^{i}-{\hat{x}}_{k|k,t-1}^{i}{∥}_{2}}{max\{∥{\hat{x}}_{k|k,t-1}^{i}{∥}_{2},\varepsilon_{0}\}}\leq \zeta ,\;\;\varepsilon_{0}>0$$

If (50) holds, accept the current local iterate at every node and proceed to Step 7. Otherwise, increment the iteration index and return to Step 5. The denominator floor prevents division by zero and matches the implementation.

Step 7: After convergence, compute the local posterior covariance in (51). Here, the matrix Young inequality bounds the unavailable cross term between the local prediction-error component and the covariance-weighted consensus-target component. The measurement noise is assumed uncorrelated with the prior prediction error and the consensus-target error, consistent with (3).

(51) $$\begin{array}{c} A_{i}=I-K_{i}\left(S_{k}^{i}+{\tilde{\Omega }}_{c,k}^{i}\right),\;B_{i}=K_{i}{\tilde{\Omega }}_{c,k}^{i},\\ P_{k|k}^{i,loc}=\left(1+\epsilon_{i}\right)A_{i}P_{k|k-1}^{i}A_{i}^{T}+K_{i}{\overset{‾}{S}}_{i}K_{i}^{T}+\left(1+\epsilon_{i}^{-1}\right)B_{i}\Lambda_{k}^{i}B_{i}^{T} \end{array}$$

Under these assumptions, $P_{k|k}^{i,loc}$ in (51) provides a conservative upper bound on the local estimation-error covariance. Here, $\Lambda_{k}^{i}$ is the covariance-intersection upper bound associated with the covariance-weighted prediction target rather than its exact covariance, ${\overset{‾}{S}}_{i}$ denotes the weighted measurement-noise contribution defined in (52), and $\epsilon_{i}>0$ is the Young parameter selected by trace balancing. Therefore, the unknown local-consensus cross-covariance is bounded rather than assumed to be zero.

(52) $${\overset{‾}{S}}_{i}=\sum_{j\in J_{i}}{\left(H_{k}^{j}\right)}^{T}{\left(B_{R,k}^{j}\right)}^{-T}{\left(\Omega_{y,I,k}^{j}\right)}^{2}{\left(B_{R,k}^{j}\right)}^{-1}H_{k}^{j}$$

Step 8: Exchange the local posterior state and covariance with neighboring nodes and apply (38a,b). The CI covariance is positive definite for positive-definite local covariances and is used in the next prediction step.

**Remark 1.** 
*DMCKF-W-MCC* [10] *applies correntropy weighting to neighboring information, whereas the proposed criterion explicitly separates the dimension-normalized innovation and consensus-disagreement residuals. Posterior CI then provides a consistent fusion mechanism when distributed posterior cross-covariances are unavailable. When the consensus strength is zero, the local IW-CWT update reduces exactly to the local DMCKF update; hence the comparison with DMCKF-W is a nested contribution test for the added consensus-information term.*

## 4. Performance Analysis

### 4.1. Consensus and Covariance Consistency Analysis

Absolute equality among all node estimates is generally not expected in the presence of heterogeneous observations, communication noise, and packet loss. The relevant performance quantity is therefore the inter-node disagreement, measured in this work by the network consensus RMS. The local IW-CWT term penalizes disagreement with the covariance-weighted prediction target, while the posterior CI stage performs a second information-space fusion of the local posterior estimates.

Assume that every local posterior covariance is consistent, meaning that it upper-bounds the corresponding local estimation-error covariance. For any nonnegative CI weights that sum to one, the fused covariance in (38a) remains a consistent bound for arbitrary and unknown correlations among the local posterior errors [8,11]. Positive definiteness of the local covariances also makes the information sum in (38a) positive definite, so the fused covariance is well defined.

**Proposition 1** (CI consistency and consensus tendency)**.** 
*Under a connected communication graph, positive normalized CI weights, and consistent local posterior covariances, repeated neighborhood fusion maps each node to a convex information combination of the available local posteriors. The fused covariance remains consistent without independence assumptions, and the disagreement vanishes when the local posterior information pairs coincide. This result establishes the covariance-consistency property required by the distributed recursion; the rate at which the states approach one another depends on the graph and the selected weights.*

### 4.2. Computational and Communication Complexity Analysis

Table 1 summarizes the dominant per-node, per-sampling-step computational and communication orders. Let *p* be the state dimension, *q* the measurement dimension, *Q* the average number of successfully received neighbors, *t* the number of fixed-point iterations, and $L_{i}=p+(Q+1)q$ the augmented innovation dimension. Dense matrix inversion and Cholesky factorization are treated as cubic operations. The entries report leading orders rather than fragile exact scalar-operation polynomials; the implementation-specific FLOPs, CPU time, communication scalars, and working memory are reported separately in Table 1.

The exchange count denotes neighborhood communication rounds per sampling step. DMCKF-IW-CWT uses one exchange of predicted information for the consensus target and a second exchange of local posterior state-covariance pairs for CI fusion. Consequently, its leading order remains polynomial in *p* and linear in *Q*, while its constant computational and communication costs exceed those of DMCKF-W.

For the conventional Kalman filter, the dominant order is:

(53) $$C_{KF}=O\left(p^{3}+p^{2}q+pq^{2}+q^{3}\right)$$

For an MCKF with *t* fixed-point iterations, the dominant order is:

(54) $$C_{MCKF}=O\;\left(t\left[p^{3}+p^{2}q+pq^{2}+q^{3}\right]\right)$$

The distributed average-consensus variant additionally processes neighboring information:

The dominant order of DMCKF-A is:

(55) $$C_{DMCKF-A}=O\left(t\left[p^{3}+\left(Q+1\right)p^{2}q\right]+Qp\right)$$

The dominant order of DMCKF-W, including posterior covariance-intersection fusion, is:

(56) $$C_{DMCKF-W}=O\left(t\left[p^{3}+\left(Q+1\right)p^{2}q\right]+Qp^{3}\right)$$

For DMCKF-IW-CWT, the consensus-information construction and posterior CI give:

(57) $$C_{DMCKF-IW-CWT}=O\;\left(t\left[p^{3}+\left(Q+1\right)p^{2}q+p^{2}\right]+Qp^{3}\right)$$

Equations (53)–(57) show that the proposed algorithm remains polynomial in *p* and linear in the average neighborhood size *Q*. The additional consensus-information construction and posterior CI exchange increase computation and communication relative to DMCKF-W, but the five-node implementation remains at the millisecond level and is faster than DMCKF-W-MCC, as reported in Table 7.

### 4.3. Fixed-Point Convergence Analysis

**Proposition 2** (fixed-point convergence with separate bandwidths)**.** 
*For a fixed node and sampling instant, consider the mapping in (26) on the closed ball (58). Assume that the prior and measurement covariances are uniformly positive definite; the augmented model, data vector, whitening factors, and covariance-weighted consensus target are bounded; and the numerical kernel floor in (27a) satisfies* $\;0<\varepsilon_{g}\;<1$ *. Define the mapping and constants in (59) and (60). If (61) and (62) hold, then the mapping has a unique fixed point in the ball and the predicted-state initialization converges to it.*

The closed ball and fixed-point map are:

(58) $$B_{i,k}\left(r\right)=\{x\in R^{p}:∥x-{\hat{x}}_{k|k-1}^{i}{∥}_{2}\leq r\}$$

(59) $$F_{i,k}\left(x\right)=A_{i,k}^{-1}\left(x\right)b_{i,k}\left(x\right)$$

In (60), *μ* is the uniform lower eigenvalue bound, the two *L* constants are Lipschitz bounds, and the final constant bounds the right-hand-side vector. Appendix A shows that the Lipschitz bounds contain terms inversely proportional to the innovation and consensus bandwidths. Therefore, moderate bandwidths increase the contraction margin. The invariance and contraction conditions are:

(60) $$\begin{array}{c} \mu ={min}_{x\in B_{i,k}\left(r\right)}\lambda_{min}\left(A_{i,k}\left(x\right)\right)>0,\\ ∥A_{i,k}\left(x\right)-A_{i,k}\left(y\right){∥}_{2}\leq L_{A}∥x-y{∥}_{2},\;∥b_{i,k}\left(x\right)-b_{i,k}\left(y\right){∥}_{2}\leq L_{b}∥x-y{∥}_{2},\\ B_{b}={max}_{x\in B_{i,k}\left(r\right)}∥b_{i,k}\left(x\right){∥}_{2}. \end{array}$$

(61) $$B_{r}={max}_{x\in B_{i,k}\left(r\right)}∥b_{i,k}\left(x\right)-A_{i,k}\left(x\right){\hat{x}}_{k|k-1}^{i}{∥}_{2}\leq \mu r$$

(62) $$\eta =\frac{L_{b}}{\mu }+\frac{L_{A}B_{b}}{\mu^{2}}<1$$

Under (61), the mapping sends the closed ball into itself; under (62), it is a contraction. Banach’s fixed-point theorem gives existence, uniqueness, and convergence. Appendix A provides the full derivation and the a posteriori residual-error bound.

The formal parameter study evaluated innovation bandwidths 2, 4, and 6; consensus bandwidths 1, 2, 4, and 6; and stopping thresholds 10^−3^ and 10^−5^, using 150 paired Monte Carlo runs for each configuration. No divergence or fixed-point nonconvergence was observed. Tightening the threshold from 10^−3^ to 10^−5^ changed the MAE by less than 10^−6^ while increasing the average iteration count by approximately one; therefore, 10^−3^ provides a favorable accuracy-efficiency balance.

### 4.4. Conditional Mean-Square Boundedness and Scope of the Stability Result

**Proposition 3** (conditional network mean-square boundedness)**.** 
*After convergence of the local fixed-point update, suppose the coupled local posterior error admits (63). The block transition matrices describe how previous errors at available nodes influence the local posterior, while the aggregate disturbance contains the process, measurement, linearization, and bounded kernel-floor terms. If the uniform row-sum, disturbance, and covariance bounds in (64) hold, and if a positive Young parameter can be selected such that (65) holds, then the distributed estimates are uniformly mean-square bounded according to (66):*

(63) $$e_{k|k}^{i,loc}=\sum_{j\in J_{i}}\Phi_{ij,k}e_{k-1|k-1}^{j}+\xi_{i,k}$$

(64) $${max}_{i}\sum_{j\in J_{i}}∥\Phi_{ij,k}{∥}_{2}\leq \bar{\phi },\;E∥\xi_{i,k}{∥}_{2}^{2}\leq \bar{q},\;p_{min}I\leq P_{k|k}^{j,loc},P_{k|k}^{i}\leq p_{max}I$$

(65) $$\kappa_{P}=\frac{p_{max}}{p_{min}},\;\;\rho =\kappa_{P}^{2}\left(1+\delta \right){\bar{\phi }}^{2}<1,\;\delta >0$$

(66) $$E_{k}\leq \rho^{k}E_{0}+\frac{\kappa_{P}^{2}\left(1+\delta^{-1}\right)\bar{q}}{1-\rho },\;\;E_{k}={max}_{i}E∥e_{k|k}^{i}{∥}_{2}^{2}$$

Appendix B proves the result using the network-coupled recursion rather than treating neighboring estimation errors as an exogenous bounded disturbance. The theorem is deliberately conditional: it does not claim unconditional asymptotic stability for arbitrary nonlinear, descriptor, fractional-order, switching-topology, or unbounded impulsive-noise systems. The nonlinear and impulsive-noise tests in Section 5 are numerical robustness and extensibility evidence.

## 5. Simulation Analysis

### 5.1. Statistical Protocol and Benchmark Configuration

**Figure 1:**
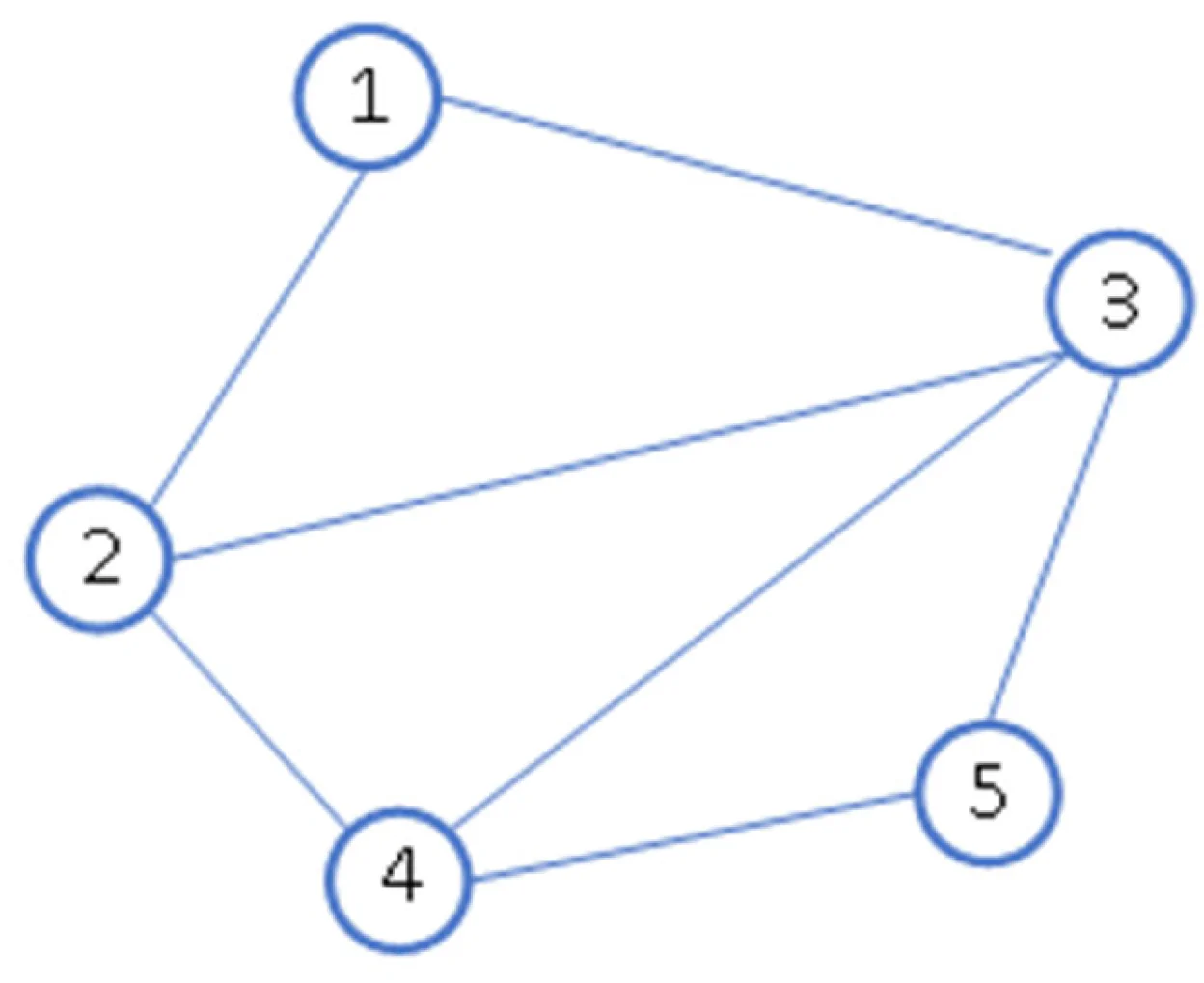
Topology of the sensor network. The numbers 1–5 denote the sensor-node indices, and the connecting lines represent communication links between neighboring nodes.

A five-node sensor network is first used as the principal benchmark. Its connected topology is shown in Figure 1. All filters are evaluated with identical true trajectories, process noise, measurement noise, packet-loss masks, and communication-noise realizations within each paired Monte Carlo run.

The two-dimensional state and scalar measurements are generated by

(67) $$x_{k}=[\begin{array}{c} \cos(\theta )\\ \sin(\theta ) \end{array}\begin{array}{c} \;-\sin(\theta )\\ \;\;\;\cos(\theta ) \end{array}]x_{k-1}+w_{k,k-1}$$

(68) $$y_{k}^{i}=[1\;\;1]x_{k}+v_{k}^{i}$$

where $\theta =\frac{\pi }{18}$ and i=1,…,5.

The process and contaminated-Gaussian measurement noises are

(69) $$w_{k,k-1}∼N\left(0,10^{-3}I_{2}\right)$$

(70) $$v_{k}^{i}∼0.9N\left(0,5\right)+0.1N\left(0,500\right)$$

The strengthened benchmark uses 500 independent Monte Carlo runs and 1000 sampling steps per run. The first 20% of the horizon is reported as the transient interval and the remaining 80% as the steady-state interval. In addition to MAE and RMSE, the evaluation includes paired Student’s *t* 95% confidence intervals, consensus RMS, normalized ANEES, 95% covariance coverage, fixed-point nonconvergence, divergence, CPU time, communication load, FLOPs, and working memory.

The initial state and initial estimate are both [1,1]^T^, and the initial covariance is the two-dimensional identity matrix. The formal benchmark uses innovation and consensus bandwidths of 6 and 2, a consensus strength of 0.05, a kernel floor of 10^−12^, at most 50 fixed-point iterations, and a stopping threshold of 10^−5^. These values were specified before the formal paired test; the separate sensitivity study assesses robustness rather than retrospectively retuning the benchmark.

### 5.2. Accuracy, Transient Convergence, and Node Agreement

**Figure 2:**
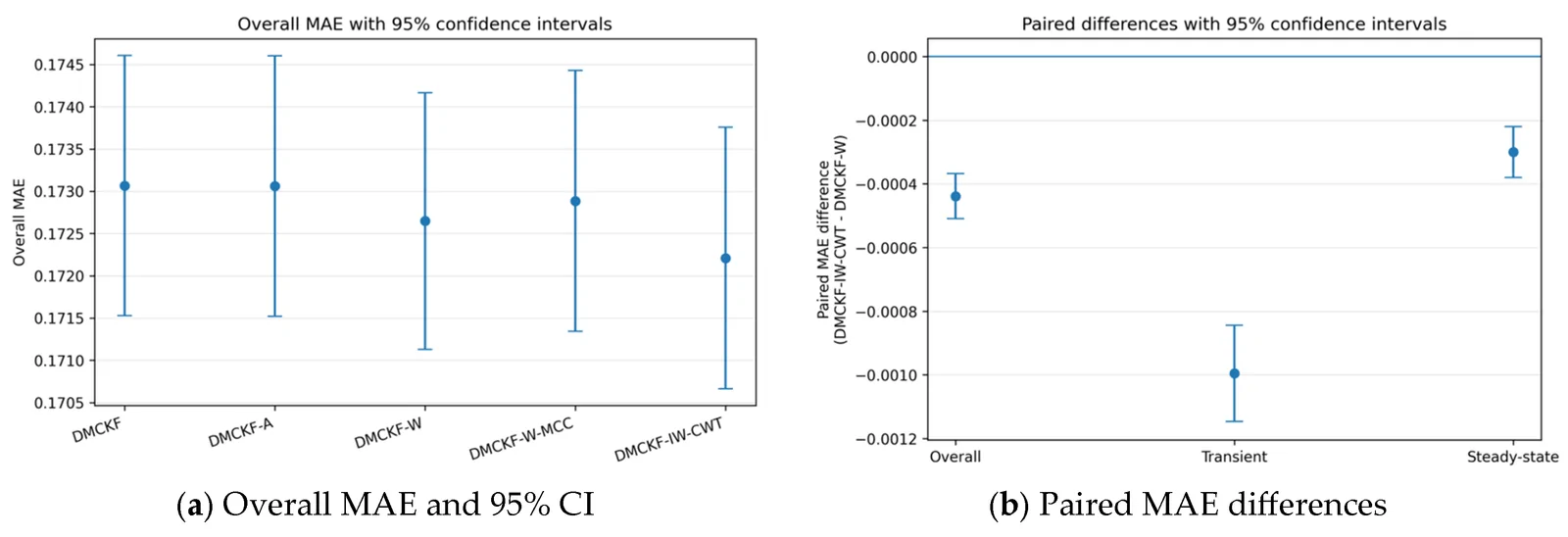
Statistical comparison in the strengthened five-node benchmark: (**a**) overall MAE with 95% confidence intervals; (**b**) paired MAE differences between DMCKF-IW-CWT and DMCKF-W, where negative values indicate improvement.

Figure 2 and Table 2 compare DMCKF, DMCKF-A, DMCKF-W, DMCKF-W-MCC, and DMCKF-IW-CWT. The error bars report 95% confidence intervals, while the paired-difference plot uses the common-random-number pairing against DMCKF-W.

DMCKF-IW-CWT achieves the lowest X1, X2, overall, transient, and steady-state MAEs among the five filters. Relative to DMCKF-W, the overall, transient, and steady-state MAEs decrease by 0.254%, 0.526%, and 0.178%, respectively, and the consensus RMS decreases by 5.371%. The paired differences are −0.000439 ± 0.000071, −0.000996 ± 0.000151, and −0.000299 ± 0.000080, respectively; all corresponding 95% confidence intervals remain below zero. The normalized ANEES changes from 1.244856 to 1.236409 and the 95% covariance coverage increases from 0.920621 to 0.921610, indicating that the accuracy improvement is obtained without degrading covariance consistency.

Although the numerical gains over DMCKF-W are moderate, the purpose of this comparison is not to claim a fundamentally different filtering framework. Rather, it isolates the effect of adding the dimension-normalized consensus-disagreement residual and posterior CI fusion under common random realizations. The statistically significant paired differences support the proposed term as a targeted and theoretically consistent extension of existing DMCKF variants.

### 5.3. Robustness to Noise and Communication Imperfections

**Figure 3:**
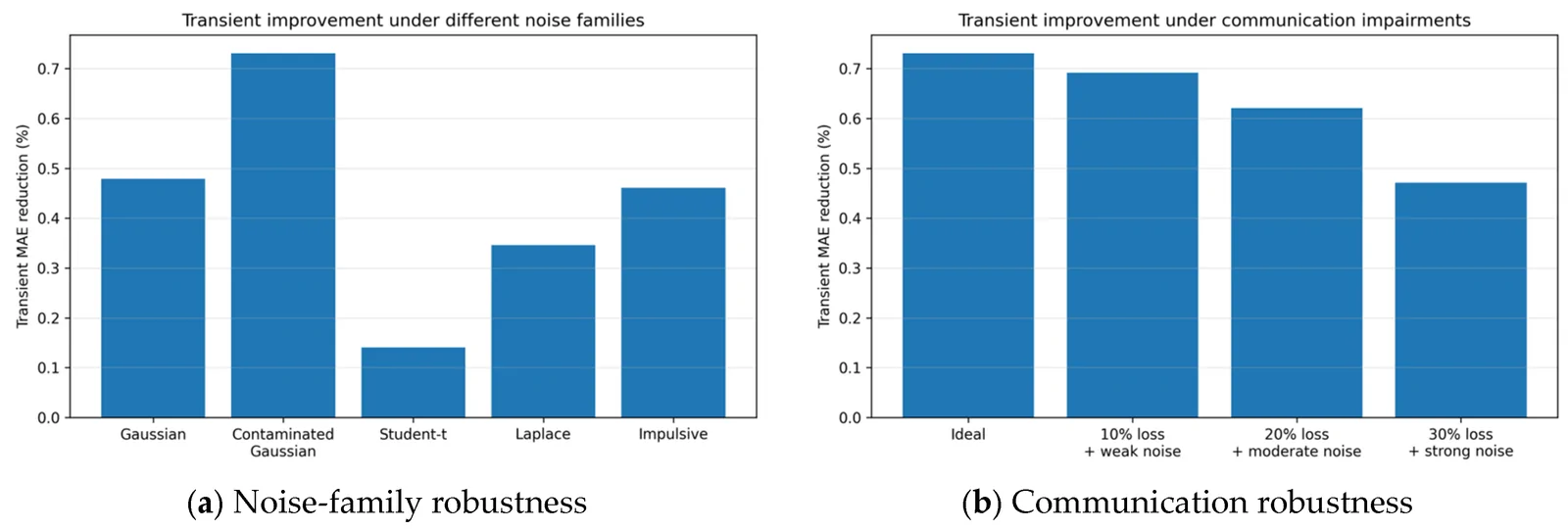
Relative transient-MAE reduction of DMCKF-IW-CWT with respect to DMCKF-W under (**a**) different noise families and (**b**) communication impairments.

The robustness study uses five measurement-noise families (Gaussian, contaminated Gaussian, Student’s *t* with three degrees of freedom, Laplace, and impulsive noise) and four communication conditions ranging from ideal links to 30% packet loss with communication noise. Figure 3 reports the relative reduction in transient MAE. Across all nine conditions, the proposed method provides a lower transient MAE than DMCKF-W, and the paired 95% confidence interval of each transient difference remains below zero. The corresponding quantitative transient-MAE and consensus-RMS improvements are summarized in Table 3.

The largest transient reduction is obtained for contaminated Gaussian noise (0.731%), which is the principal non-Gaussian benchmark. The consensus RMS is also reduced in every noise and communication case. Under 10% packet loss, the overall, transient, and steady-state MAEs are all lower than those of DMCKF-W. Even at 30% packet loss with stronger communication noise, the transient MAE remains lower, showing that the consensus-weighted mechanism continues to accelerate early convergence under severe link impairments.

### 5.4. Parameter Sensitivity and Fixed-Point Convergence

All 24 combinations of the three innovation bandwidths, four consensus bandwidths, and two stopping thresholds completed without divergence or fixed-point nonconvergence. The complete 24-configuration sweep indicates that the innovation bandwidth has a stronger influence on the MAE than the consensus bandwidth. Table 4 presents representative configurations that illustrate the sensitivity to the consensus bandwidth and stopping threshold. Tightening the threshold from 10^−3^ to 10^−5^ leaves the MAE unchanged to six decimal places but increases the mean number of iterations from 2.118 to 3.111. This supports the use of 10^−3^ when lower computational latency is preferred.

### 5.5. Nonlinear and Higher-Dimensional Validation

A four-state constant-velocity model observed by eight nonlinear range sensors was evaluated for 300 Monte Carlo runs of 600 steps under Student’s *t* noise, 10% packet loss, and communication noise. DMCKF-IW-CWT completed all runs with zero divergence and reduced the consensus RMS from 0.033556 to 0.032505. This experiment confirms that the local linearization interface can be combined with the proposed consensus-weighted update and posterior CI fusion without changing the overall algorithmic structure. The corresponding nonlinear validation results are summarized in Table 5.

This nonlinear test is used as numerical extensibility evidence rather than as a claim of unconditional nonlinear stability. It shows that the same local linearization interface, consensus-disagreement term, and posterior CI fusion can be applied without changing the overall algorithmic structure, while the sufficient convergence and boundedness conditions in Propositions 2 and 3 still need to be checked for each concrete nonlinear model. The representative higher-dimensional scalability results are summarized in Table 6.

For the representative four- and eight-state networks in Table 6, the proposed method reduces the overall MAE by 0.424–0.777%, the transient MAE by 1.768–2.188%, and the consensus RMS by 3.698–4.849%. The paired 95% confidence intervals for the overall and transient differences remain below zero in these cases. These results show that the benefit of the consensus-weighted update is retained as the state dimension and network size increase.

### 5.6. Measured Computational and Communication Cost

Table 7 shows that the consensus-weighted local update and posterior CI fusion introduce additional computation and one additional exchange of posterior state-covariance information. Nevertheless, the average execution time remains 2.277 ms per step, and the working memory is 0.309 MB in the five-node benchmark. The proposed implementation is faster than DMCKF-W-MCC (2.792 ms per step) and provides the lowest MAE and consensus RMS in Table 2. Thus, the added cost remains compatible with millisecond-level numerical processing and is exchanged for statistically significant accuracy and consensus improvements.

## 6. Conclusions

This paper proposes DMCKF-IW-CWT for distributed state estimation under non-Gaussian noise. The method separates the whitened innovation and consensus-disagreement residuals in a dimension-normalized correntropy objective, embeds them in a fixed-point local update, and applies posterior covariance intersection without requiring neighboring posterior cross-covariances. A sufficient contraction-based convergence condition is provided under (58)–(62), and a network-coupled conditional mean-square boundedness result is established under (63)–(66). In the strengthened five-node benchmark, the overall, transient, and steady-state MAEs decrease by 0.254%, 0.526%, and 0.178% relative to DMCKF-W, and the consensus RMS decreases by 5.371%; the paired 95% intervals of the three MAE differences remain below zero. The transient MAE and consensus RMS are also reduced across the tested noise and communication conditions, and representative four- and eight-state networks retain significant overall and transient improvements. The posterior CI stage introduces additional computation and communication, but the measured execution remains at the millisecond level. The theoretical claims are restricted to Propositions 2 and 3; the nonlinear and impulsive-noise experiments are numerical validations rather than proofs of unconditional asymptotic stability.

## Figures and Tables

**Table 1:** Dominant per-step computational and communication orders.

Algorithm	Local Filtering Term	Network-Dependent Term	Dominant Total Order	Neighborhood Exchanges
KF	O(*p*^3^ *+ p*^2^*q + pq*^2^ *+ q*^3^)	/	O(*p*^3^ + *p*^2^*q* + *pq*^2^ + *q*^3^)	0
MCKF	O(*t*[*p*^3^ *+ p*^2^*q + pq*^2^ *+ q*^3^])	/	O(*t*[*p*^3^ + *p*^2^*q* + *pq*^2^ + *q*^3^])	0
DMCKF-A	O(*t*[*p*^3^ + (*Q* + 1)*p*^2^*q*])	O(*Qp*)	O(*t*[*p*^3^ + (*Q* + 1)*p*^2^*q*] + *Qp*)	1
DMCKF-W	O(*t*[*p*^3^ + (*Q* + 1)*p*^2^*q*])	O(*Qp*^3^)	O(*t*[*p*^3^ + (*Q* + 1)*p*^2^*q*] + *Qp*^3^)	1
DMCKF-IW-CWT	O(*t*[*p*^3^ + (*Q* + 1)*p*^2^*q* + *p*^2^])	O(*Qp*^3^)	O(*t*[*p*^3^ + (*Q* + 1)*p*^2^*q* + *p*^2^] + *Qp*^3^)	2

**Table 2:** Accuracy and consensus performance in the strengthened benchmark.

Filter	X1 MAE	X2 MAE	Overall MAE	Consensus RMS
DMCKF	0.178894	0.167243	0.173068	0.116240
DMCKF-A	0.178887	0.167237	0.173062	0.114074
DMCKF-W	0.178508	0.166789	0.172649	0.010048
DMCKF-W-MCC	0.178697	0.167076	0.172886	0.120418
DMCKF-IW-CWT	0.178029	0.166391	0.172210	0.009508

**Table 3:** Transient and consensus improvements relative to DMCKF-W.

Test Condition	Consensus RMS Reduction (%)	Reduction (%)	Transient MAE IW-CWT
Gaussian	5.291	0.479	0.158789
contaminated Gaussian	5.331	0.731	0.202626
Student-t	5.405	0.141	0.151154
Laplace	5.361	0.346	0.155639
impulsive noise	5.305	0.461	0.164828
ideal communication	5.331	0.731	0.202626
10% packet loss	4.121	0.692	0.204810
20% packet loss	2.947	0.621	0.205626
30% packet loss	1.448	0.471	0.207380

**Table 4:** Representative bandwidth and stopping-threshold configurations selected from the 24 tested combinations; each configuration uses 150 paired Monte Carlo runs.

Innovation Bandwidth $\sigma_{I}$	Consensus Bandwidth $\sigma_{c}$	Stopping Threshold ξ	Overall MAE	Mean Fixed-Point Iterations
6	1	$10^{-3}$	0.180353	2.128
6	2	$10^{-3}$	0.180295	2.118
6	4	$10^{-3}$	0.180279	2.117
6	6	$10^{-3}$	0.180276	2.116
6	2	$10^{-5}$	0.180295	3.111

**Table 5:** Nonlinear range-only validation under Student’s *t* noise, 10% packet loss, and communication noise.

Model	Noise and Communication Setting	Runs	Divergence	Consensus RMS of DMCKF-W	Consensus RMS of DMCKF-IW-CWT	Main Observation
4-state constant-velocity target with 8 range sensors	Student-t noise (df = 3), 10% packet loss, and additive communication noise	300 Monte Carlo runs, 600 steps	0/300	0.033556	0.032505	The consensus RMS is reduced by 3.13%, and no fixed-point divergence is observed.

**Table 6:** Representative higher-dimensional scalability results.

States/Nodes	Overall MAE DMCKF-W	Overall MAE IW-CWT	Overall Reduction (%)	Transient Reduction (%)	Consensus Reduction (%)
4/5	0.109969	0.109115	0.777	2.188	3.698
4/10	0.096852	0.096404	0.463	1.768	4.849
8/10	0.105467	0.104736	0.693	2.187	4.643
8/20	0.096201	0.095793	0.424	1.868	4.745

**Table 7:** Measured computational, communication, and memory cost per step.

Filter	CPU Time (ms)	Communication Scalars	Approx. FLOPs	Memory (MB)
DMCKF	1.010	14	1508.9	0.252
DMCKF-A	1.066	84	1676.8	0.252
DMCKF-W	1.248	84	1749.9	0.252
DMCKF-W-MCC	2.792	84	2718.1	0.286
DMCKF-IW-CWT	2.277	154	2996.2	0.309

## Data Availability

The simulation data used in this study are available from the corresponding author upon reasonable request.

## Appendix A. Proof of Proposition 2

**Proof of Proposition 2. ** 
For readability, node and time indices are suppressed where no ambiguity arises. The predicted state is denoted by a superscript minus. The closed ball, mapping, and constants are defined in (58)–(60). First, consider the original Gaussian kernel and its numerically stabilized counterpart defined in (27a). The derivative of the original Gaussian kernel satisfies $\left|G_{\sigma }^{\prime }\left(u\right)\right|\leq \frac{1}{\sigma \sqrt{e}}$. Moreover, the lower-clipping operator $x\to max\{x,\varepsilon_{g}\;\}$ is nonexpansive. Therefore, the stabilized kernel ${\overset{‾}{G}}_{\sigma }\;(\cdot )$ is also globally Lipschitz, with a Lipschitz constant no larger than $\frac{1}{\sigma \sqrt{e}}$, as summarized in (A1):

(A1) $$\begin{array}{c} G_{\sigma }\left(u\right)=\exp\left[-u^{2}/\left(2\sigma^{2}\right)\right],\\ {\overset{‾}{G}}_{\sigma }\;\left(u\right)=\max\left\{G_{\sigma }\left(u\right),\varepsilon_{g}\right\},\;\;{0<\varepsilon }_{g}<1,\\ \left|G_{\sigma }^{\prime }\left(u\right)\right|=\frac{\left|u\right|}{\sigma^{2}}\exp\left[-u^{2}/\left(2\sigma^{2}\right)\right]\leq \frac{1}{\sigma \sqrt{e}}\\ \left|{\overset{‾}{G}}_{\sigma }(u)-{\overset{‾}{G}}_{\sigma }(v)\right|\leq \left|G_{\sigma }\left(u\right)-G_{\sigma }\left(v\right)\right|\leq \frac{1}{\sigma \sqrt{e}}\left|u-v\right| \end{array}$$

For each innovation component, applying (A1) to the affine residual map gives the augmented-row norm divided by the innovation bandwidth and the square root of e. For the whitened consensus residual, the corresponding bound is proportional to the inverse whitening-factor norm divided by the consensus bandwidth and the square root of e. On the compact ball, all model and normalization factors are bounded; hence:

(A2) $$∥A\left(x\right)-A\left(y\right){∥}_{2}\leq L_{A}∥x-y{∥}_{2},\;\;∥b\left(x\right)-b\left(y\right){∥}_{2}\leq L_{b}∥x-y{∥}_{2}$$

Because the stabilized innovation and consensus weights satisfy ${\overset{‾}{G}}_{\sigma }\;(\cdot )\geq {\varepsilon \;}_{g}>0$, their diagonal weight matrices satisfy $\Omega_{I,k}^{i}\geq {\varepsilon \;}_{g}I$, $\Omega_{c,k}^{i}\geq {\varepsilon \;}_{g}I$. Together with the uniformly positive-definite covariance factors assumed in Proposition 2, this yields a strictly positive lower bound for the coefficient matrix of the fixed-point mapping. Consequently, the inverse coefficient matrix is uniformly bounded. Condition (61) gives:

(A3) $$∥F\left(x\right)-{\hat{x}}^{-}{∥}_{2}\leq ∥A^{-1}\left(x\right){∥}_{2}∥b\left(x\right)-A\left(x\right){\hat{x}}^{-}{∥}_{2}\leq \frac{B_{r}}{\mu }\leq r$$

Therefore, the fixed-point map sends the closed ball into itself. For any two points in the ball, add and subtract the intermediate term formed with the first inverse coefficient matrix and the second right-hand-side vector. Then apply the standard inverse-difference identity to obtain:

(A4) $$\begin{array}{c} F\left(x\right)-F\left(y\right)=A^{-1}\left(x\right)\left[b\left(x\right)-b\left(y\right)\right]\\ +A^{-1}\left(x\right)\left[A\left(y\right)-A\left(x\right)\right]A^{-1}\left(y\right)b\left(y\right) \end{array}$$

Applying the inverse, Lipschitz, and right-hand-side bounds gives

(A5) $$∥F\left(x\right)-F\left(y\right){∥}_{2}\leq \left(\frac{L_{b}}{\mu }+\frac{L_{A}B_{b}}{\mu^{2}}\right)∥x-y{∥}_{2}=\eta ∥x-y{∥}_{2}$$

Condition (62) makes the mapping a contraction on a complete metric space. Banach’s theorem guarantees a unique fixed point and convergence from the predicted-state initialization. The standard a posteriori estimate is:

(A6) $$∥x^{\left(t\right)}-x^{⋆}{∥}_{2}\leq \frac{\eta }{1-\eta }∥x^{\left(t\right)}-x^{\left(t-1\right)}{∥}_{2}$$

Consequently, reducing the stopping threshold decreases the residual fixed-point error, while increasing either bandwidth decreases the corresponding Gaussian-weight Lipschitz contribution. This completes the proof of Proposition 2. □

## Appendix B. Proof of Proposition 3

**Proof of Proposition 3. ** 
Let the local posterior error denote the estimation error before CI fusion. Because the consensus target depends on neighboring predictions, the correct recursion is network coupled:

(A7) $$e_{k|k}^{i,loc}=\sum_{j\in J_{i}}\Phi_{ij,k}e_{k-1|k-1}^{j}+\xi_{i,k}$$

Define the network error level as the largest node-wise mean-square error. The block row-sum condition in (64), followed by the vector Young inequality with a positive parameter, gives:

(A8) $$E∥e_{k|k}^{i,loc}{∥}_{2}^{2}\leq \left(1+\delta \right){\bar{\phi }}^{2}E_{k-1}+\left(1+\delta^{-1}\right)\bar{q}$$

The posterior CI estimate is a matrix-weighted combination of local posteriors, so its error satisfies:

(A9) $$e_{k|k}^{i}=\sum_{j\in J_{i}}M_{ij,k}e_{k|k}^{j,loc},\;\;M_{ij,k}=P_{k|k}^{i}\omega_{ij}{\left(P_{k|k}^{j,loc}\right)}^{-1}$$

The covariance bounds in (64) imply the following bound for the CI coefficient matrices:

(A10) $$\sum_{j\in J_{i}}∥M_{ij,k}{∥}_{2}\leq \kappa_{P},\;\;E∥e_{k|k}^{i}{∥}_{2}^{2}\leq \kappa_{P}^{2}{max}_{j}E∥e_{k|k}^{j,loc}{∥}_{2}^{2}$$

Combining (A8) and (A10), and using the definition of ρ in (65), yields:

(A11) $$E_{k}\leq \rho E_{k-1}+\kappa_{P}^{2}\left(1+\delta^{-1}\right)\bar{q}\;\Rightarrow \;E_{k}\leq \rho^{k}E_{0}+\frac{\kappa_{P}^{2}\left(1+\delta^{-1}\right)\bar{q}}{1-\rho }$$

Because ρ < 1, (A11) is finite uniformly in time and proves (66). No independence among node estimation errors is required; their coupling is handled by the block row-sum bound. This is a sufficient conditional result, and the effective-transition and covariance bounds must be verified for each nonlinear or time-varying application. This completes the proof of Proposition 3. □
